# Current Evidence and Expert Opinion on Thromboprophylaxis After Total Knee and Hip Replacement

**DOI:** 10.7759/cureus.51089

**Published:** 2023-12-25

**Authors:** Bharat S Mody, Manuj Wadhwa, Ronen Roy, Shwetha Echila

**Affiliations:** 1 Joint Replacement Surgery, Welcare Hospital, Vadodara, IND; 2 Orthopaedics & Joint Replacement, Elite Institutes of Orthopaedics & Joint Replacement, Mohali, IND; 3 Orthopaedic Surgery, Fortis Hospitals, Kolkata, IND; 4 Internal Medicine, Pfizer, Mumbai, IND

**Keywords:** venous thromboembolism (vte), total knee replacement (tkr), total hip replacement (thr), novel oral anticoagulants, low molecular weight heparin (lmwh), deep vein thrombosis (dvt)

## Abstract

An effective anticoagulant provides a balance between the risk for venous thromboembolism (VTE) and bleeding and is crucial in achieving optimal clinical outcomes in patients undergoing total hip replacement (THR) and total knee replacement (TKR) surgeries. We performed a review of the literature on thromboprophylaxis for patients undergoing total hip or knee replacement. This review article summarizes current guidelines and evidence for anticoagulation along with the expert opinion about pharmacological VTE prophylaxis, particularly non-Vitamin K antagonist oral anticoagulants (NOACs), for patients after total hip or knee replacement. Aspirin for VTE prophylaxis after TKR/THR has been controversial and most of the evidence is reported from observational research. Although the guidelines do not recommend any specific thromboprophylaxis agent, available evidence suggests that NOACs are as effective as low molecular weight heparins (LMWHs) in preventing VTE. Oral administration and the lack of dose monitoring make NOACs easy to use in outpatient settings in cases with challenging treatment compliances. They can be used for two weeks after TKR and five weeks after THR - six weeks after TKR and THR to cover the at-risk period for VTE post-discharge. Owing to the lack of evidence for a head-to-head comparison of NOACs, an anticoagulant with better efficacy and safety may be suggested in special patient populations (elderly, obese patients, or those with renal dysfunction). The expert opinion on pharmacological VTE prophylaxis provided in this article could address some gaps in the management of anticoagulation in patients with total hip or knee replacement.

## Introduction and background

**Figure 1:**
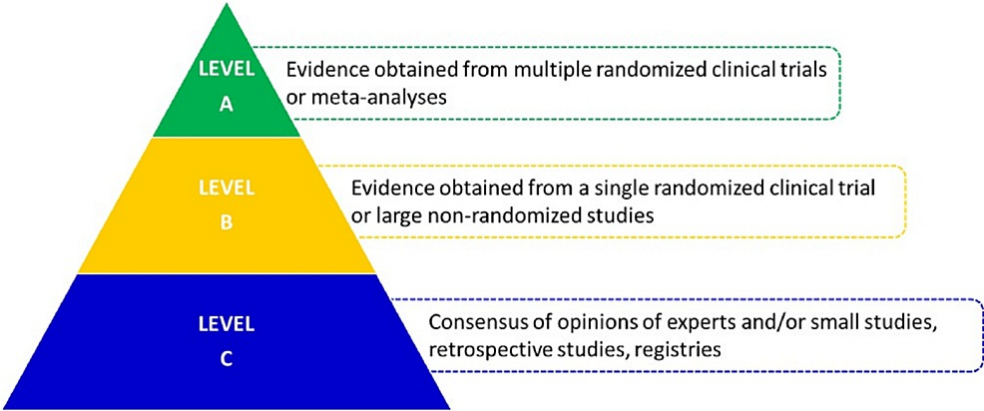
Levels of evidence - European Society of Cardiology Adapted from Ponikowski P et al. [13]

Total knee replacement (TKR) and total hip replacement (THR) are the two most common surgeries performed to improve the quality of life and reduce morbidity in patients with arthritis of the knee and hip, respectively. As per the estimate from a national registry, there is a steady increase in the number of TKRs reported to the registry from 1,019 in 2006 to around 27,000 in 2019 [1]. These patients are at an increased risk of developing venous thromboembolism (VTE) which includes deep vein thrombosis (DVT) and pulmonary embolism (PE). VTE is an important cause of long-term morbidity and also represents a preventable cause of mortality with substantial healthcare costs [2]. Current clinical practice guidelines in orthopedic surgery recommend both mechanical and pharmacological interventions for VTE prophylaxis [3]. The American Academy of Orthopaedic Surgeons (AAOS) guidelines were last released almost a decade ago and have not been updated as per recent evidence. Studies have shown that without pharmacologic thromboprophylaxis, rates of DVT are in the order of 54% after THR and 64% after TKR [4-8]. Most VTE events occur during the post-discharge follow-up period, which is often not recorded in India [9-11]. When most patients experience a VTE event, they consult either a cardiovascular specialist or a physician. In such a situation, VTE might go unnoticed by an orthopedic surgeon. Therefore, it becomes imperative to use an effective anticoagulant to prevent a VTE without significantly increasing the risk of bleeding. This article aims to review current guidelines and recent evidence about pharmacological VTE prophylaxis and provides expert opinion based on clinical experience that could fill the existing gaps [12]. Levels of evidence have been assessed using methods adapted by the European Society of Cardiology (ESC) (Figure 1) [13].

## Review

VTE prophylaxis before the non-Vitamin K antagonist oral anticoagulants (NOAC) era

Traditionally, low-molecular-weight heparins (LMWH) and vitamin K antagonists (VKAs) were thromboprophylactic agents extensively used after THA and TKA.

Limitations of Traditional Agents

LMWH and fondaparinux are inconvenient in outpatient settings because of their parenteral administration and high cost. Heparin-induced thrombocytopenia is an additional risk with LMWHs. Fondaparinux carries additional limitations as it is not recommended for patients <50 kg or aged >75 years, and those with moderate or severe renal impairment [3].

VKAs have a slow onset and offset of actions, and a narrow therapeutic window with a target internationalized normalized ratio of two to three. Its complex pharmacodynamics requires frequent monitoring of coagulation and dose adjustment to achieve therapeutic levels and often uses additional resources, thus causing potential confusion for patients [14-16]. There is a propensity for interaction with other drugs and various foods based on their vitamin K content, and its metabolism is vulnerable to numerous genetic polymorphisms. Please refer to Table 1 [17].

**Table 1:** Limitations of conventional anticoagulants [17] ASH: American Society of Hematology; LMWH: low molecular weight heparin; SIGN: Scottish Intercollegiate Guidelines Network; VKA: vitamin-K antagonist

Parenteral Anticoagulants
Inconvenient for out-patient settings
High cost
LMWH – Risk of heparin-induced thrombocytopenia
Fondaparinux – Not recommended for patients <50 kg or aged >75 years, and those with moderate or severe renal impairment
Oral Anticoagulants
VKA
Slow onset and offset of actions
Narrow therapeutic window
Complex pharmacodynamics requires frequent monitoring
Interaction with other drugs and various foods
Aspirin
No clarity regarding dosage and duration of administration
Conditional recommendation by 2019 ASH guidelines
SIGN guidelines do not recommend aspirin as monotherapy

Current guidelines

**Figure 2:**
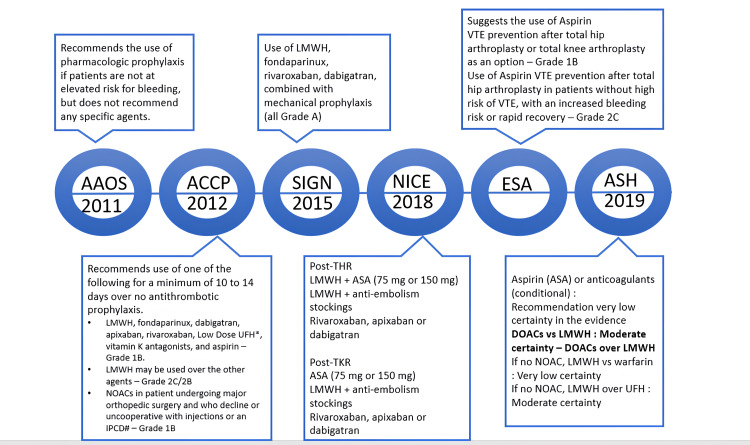
Evolution of VTE prophylaxis guidelines [18-23] AAOS: The American Academy of Orthopaedic Surgeons; ACCP: American College of Chest Physicians; ASA: Aspirin; ASH: American Society of Hematology; DOAC: direct-acting oral anticoagulants; ESA: European Society of Anaesthesiology; LMWH: low molecular weight heparins; NICE: National Institute for Health and Care Excellence; NOACs: non-Vitamin K antagonist oral anticoagulants; SIGN: Scottish Intercollegiate Guidelines Network; THR: total hip replacement; TKR: total knee replacement; UFH: unfractionated heparin; VTE: venous thromboembolism.

Several orthopedic associations have published evidence-based guidelines for managing VTE prophylaxis in major orthopedic surgeries. The VTE regimens recommended by various guidelines are summarized in Figure 2 [18-23].

Clinical Practice Guidelines and the Aspirin Controversy

Aspirin (acetylsalicylic acid) is an inexpensive, orally administered, and widely available medication with a controversial use as a prophylactic agent for VTE. The orthopedic community has long embraced aspirin for postsurgical VTE prophylaxis, mainly after total hip arthroplasty (THA) and total knee arthroplasty (TKA). This was due to a noticeable increase in perioperative bleeding complications after introducing fractionated heparins for VTE prophylaxis in total joint replacement. This was majorly based on aspirin's perceived low bleeding risk without any convincing evidence. Indeed, the majority of the observational data is set back to the time (2000-2017) when orthopedic surgeons favored aspirin prophylaxis, except for patients who were perceived to be at a higher risk who then selectively received warfarin or other anticoagulants [24-26]. Moreover, they have not looked at pulmonary embolism (PE) - a more reliable surrogate marker of VTE [27-30]. Results from the pulmonary embolism prevention after hip and knee replacement (PEPPER) study may help address the issue. PEPPER is a phase IV study that compares aspirin, warfarin, and rivaroxaban for patients undergoing TKR or THR. Recently published 2019 American Society of Hematology (ASH) guidelines included conditional recommendations for using aspirin. This guideline also recommended preferring NOACs over LMWH which is preferred over unfractionated heparin (UH) for patients undergoing total hip or knee arthroplasty [23]. There is no clarity regarding the dosage and duration of administering aspirin for VTE prophylaxis [31]. The Scottish Intercollegiate Guidelines Network (SIGN) guidelines do not recommend aspirin as a sole prophylactic agent [20]. Table 2 included expert opinions on using aspirin in patients undergoing THR or TKR [3,32-38].

**Table 2:** Expert opinion on the use of pharmacological prophylaxis for patients undergoing total hip or total knee replacement NOAC: novel oral anticoagulants, VTE: venous thromboembolism prophylaxis; PCC: prothrombin complex concentrate; TKA: total knee arthroplasty; THA: total hip arthroplasty; LMWHs: low-molecular-weight heparin

Sr. No.			Expert Opinion	Level of Evidence
		Aspirin for VTE prophylaxis after TKR/THR [3, 32-38]		
1			The use of aspirin as a VTE prophylaxis agent in orthopedic patients undergoing THA and TKA remains controversial across the clinical practice guidelines.	C
2			Expert opinion: recent observational evidence supports using aspirin for preventing VTE in THA and TKA but is limited by selection bias favoring aspirin. High-quality evidence that compares aspirin with NOACs is limited, thus suggesting its use in selected low-risk patients and patients in whom administering NOACs is a challenge.	C
		NOACs for VTE prophylaxis after TKR/THR [4,5,10,39-62]		
3			NOACs are preferred over other standard-of-care anticoagulants; NOACs are preferred over LMWH.	B
			NOACs are preferred over warfarin.	B
			NOACs are preferred over aspirin.	B
			LMWH is preferred over warfarin.	C
			Warfarin is preferred over aspirin.	C
4			NOACs are preferred over standard-of-care for elderly patients for VTE prophylaxis after TKA/THA.	B
5			NOACs are preferred over standard-of-care for bodyweight <60 and >120kg patients for VTE prophylaxis after TKR/THR.	B
6			In patients with renal dysfunction, NOACs exhibit similar rates of VTE but lower bleeding complications compared to LMWHs, as shown by evidence from subgroup analyses of randomized trials and meta-analyses.	A
7			Apixaban may be the preferred anticoagulant as among the NOACs, apixaban has the lowest renal elimination and no dose adjustment is required in patients with renal impairment.	C
8			The approved dose and duration of different NOACs are as in Figure 2.	A
9			In patients undergoing revision surgery or surgery expected to last for more than 2 hours with extensive blood loss, NOAC with a better safety profile along with efficacy should be preferred.	C
		Challenges and practical aspects of VTE prophylaxis after TKR/THR in India		
10			Since there are no validated risk assessment models for VTE and bleeding in patients undergoing TKR or THR, VTE prophylaxis should be selected considering the balance between thrombotic and bleeding risk factors.	C
11			NOACs may be preferred for patients with a high risk of VTE.	C
12			Apixaban and dabigatran may be preferred for patients with a higher risk of bleeds.	C
13			Apixaban and dabigatran may be preferred in patients with a previous history of bleeding.	C
14			NOACs may be the preferred anticoagulants in patients with a previous history of VTE.	C
15			Combined antiplatelet and anticoagulant therapy should be used only in patients with a low risk of bleeding and a higher risk of thromboembolic disease events.	C
16			The concomitant administration of antiplatelets along with anticoagulants has to be done carefully after assessing the thrombotic and bleeding risk. If the bleeding risk is high, the antiplatelets have to be withheld till the duration of prophylaxis.	Expert Opinion
17			Initiation of anticoagulation should be avoided for at least 8 hours after the surgery. Hemostasis has to be ensured before initiating anticoagulation.	C
18			NOACs have to be stopped considering the time of the last dose and renal function.	C
19			NOACs have to be stopped before 24 hours for minor procedures and 72 hours before major surgeries.	C
20			Supportive treatment is sufficient for minor NOAC-induced bleeds. PCC can be the alternative strategy in the major bleeds caused by NOACs. Antidotes should be reserved only for severe and life-threatening bleeding.	C
21			VTE prophylaxis with the agent administered twice a day may have a lower probability of hazardously high peaks or low troughs when skipping a dose or when an overdose occurs compared to agents with once-daily dosing [63-65].	A
22			Patients have to be counseled on the signs and symptoms of VTE with the instruction to reach out to the hospital if necessary. VTE prophylaxis should be for 4 weeks after TKA and 6 weeks after THA.	C

VTE prophylaxis in the NOACs era

**Figure 3:**
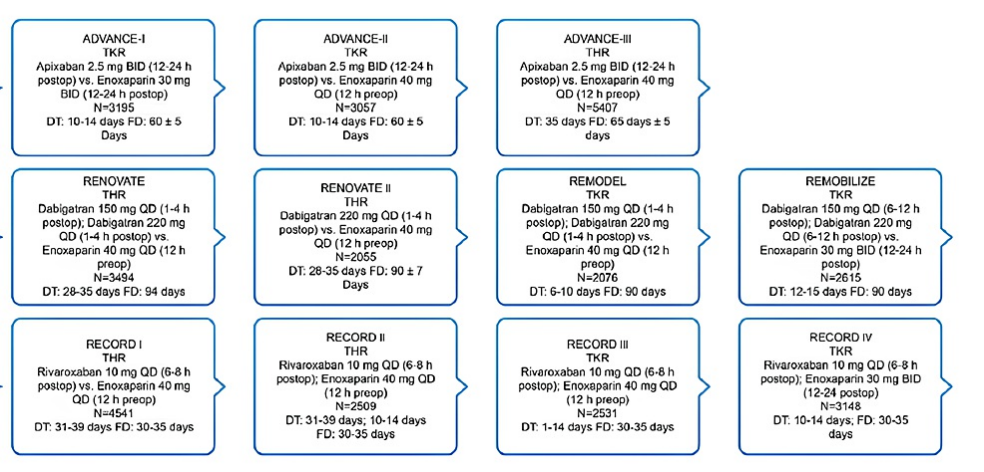
Landmark clinical trials of NOACs [17] DT: Duration of treatment, FD: follow-up duration, NOAC: non-Vitamin K antagonist oral anticoagulant; OD: once daily; THR: total hip replacement; TKR: total knee replacement; BID: twice daily

The introduction of NOACs helped address some limitations of traditional anticoagulants. A summary of NOAC pharmacology is presented in Table 3. Apixaban and rivaroxaban directly inhibit factor Xa, whereas dabigatran is a direct thrombin inhibitor [2,4,66]. NOACs have been studied in several phase III trials for preventing VTE following joint replacement (Figure 3) [17].

**Table 3:** Pharmacological profiles of apixaban, rivaroxaban and dabigatran [2, 4, 66] GI: gastrointestinal; BID: twice daily; OD: once daily; VKORC1: C1 subunit of the vitamin K epoxide reductase enzyme.

Drug	Apixaban	Rivaroxaban	Dabigatran	Warfarin
Mechanism of action	Direct factor Xa inhibitor	Direct factor Xa inhibitor	Direct thrombin Ⅱa inhibitor	VKORC1
Dosing	BID	OD	BID	OD
Bioavailability (%)	50	66% without food and up to 100% with food	6.5	100
Prodrug	No	No	Yes	No
Protein binding (%)	87	92-95	35	99
Time of peak concentration (hours)	1-4	2-4	0.5-2	4-5 days
Half-life (hours)	12	5-9 (young) 11-13 (elderly)	12-17	40
Metabolism	Oxidation (CYP3A4) and conjugation	Oxidation (CYP3A4/3A5/CYP2J2)	Conjugation	100% liver
Excretion	Renal, 27% (unchanged) and biliary, 75%	Renal 33% (unchanged), Biliary 7% (unchanged)	Renal, 84% (unchanged) and biliary, 20%	92% renal
Absorption with food	No effect	+ 39% more	No effect	Yes

Primary Efficacy and Safety Outcomes for VTE Prophylaxis in Patients With THR and TKR in Randomized Trials

Dabigatran 150 mg and 220 mg once daily were found non-inferior to enoxaparin 40 mg once daily for primary efficacy outcome for a composite of total VTE, major VTE, and all-cause death. Pooled analysis of the four phase III studies further substantiated that both doses of dabigatran had similar efficacy and safety profiles [43].

Rivaroxaban 10 mg once daily was found superior to enoxaparin for the primary efficacy endpoint of a composite of DVT, nonfatal PE, and all-cause mortality in phase III trials. Pooled analysis phase III studies confirmed the results and showed that rivaroxaban effectively reduced the incidence of symptomatic VTE and all-cause mortality [44]. However, the analysis also showed a trend towards higher risk for major and clinically relevant bleeding [49-51].

Pooled analysis of the ADVANCE trials showed apixaban 2.5 mg b.i.d was more effective than enoxaparin 40 mg once daily in preventing major VTE (p = 0.001) without an increase in bleeding [52].

In several meta-analyses, NOACs caused a reduction of VTE events, including proximal DVT, PE, and VTE-related mortality compared to LMWHs. There was no statistically significant difference between both groups in the incidence of major bleeding [53-57].

Are All NOACs the same?

No head-to-head RCTs comparing apixaban, rivaroxaban, and dabigatran have been performed; thus, results of indirect comparisons need to be interpreted with caution. While interpreting rates of major bleeding in the VTE prevention trials, it is important to note that apixaban and dabigatran trials included surgical-site bleeding in their definition of major bleeding whereas rivaroxaban trials did not. Despite this, bleeding rates have been low with apixaban compared to rivaroxaban and dabigatran (RR: 0.78 vs. 1.85 vs. 1.09, respectively), as described in the pooled analysis of phase III trials of NOACs conducted by Eriksson et al. [17]. Moreover, it is noteworthy that >50% of major bleeding events in the apixaban arm occurred before the first postoperative administration of apixaban (i.e., after surgery) in the ADVANCE studies (five/nine in ADVANCE-2; 13/22 events in ADVANCE-3). A standard network meta-analysis for fondaparinux, dabigatran, rivaroxaban, apixaban, edoxaban, and enoxaparin of 19 RCTs (N = 43 838) was performed by Hur M et al. to compare the efficacy and safety of anticoagulants for preventing VTE after hip and knee arthroplasty by calculating the surface under the cumulative ranking (SUCRA) probabilities [9]. SUCRA probabilities range from 0 to 100, which allows a simple transformation of the mean rank in cumulative ranking analysis where a higher SUCRA value means a higher rank of the treatment. In the clustered ranking plot, as per the SUCRA values, apixaban had the most favorable position for both VTE and major/clinically relevant non-major (CRNM) bleeding. In another meta-analysis, RR of major/CRNM bleeding was lowest for apixaban 2.5 mg b.i.d (0.84; 95% CI 0.70-0.99) and highest for rivaroxaban (1.27; 95% CI 1.01-1.59) and fondaparinux (1.64; 95% CI 0.24-11.35) [8]. Similarly, in a meta-analysis ranking the efficacy of NOACs, the bleeding rate was stable with apixaban whereas it remained relatively high with rivaroxaban [56,58]. The expert opinion about NOACs is presented in Table 2 [4,5,10,39-62].

Evidence for Specific Patient Subgroups/Special Population (Renal/Elderly/Obese/History of Bleeds/Duration of Surgery/Bleeding During Surgery/Revision/Previous History of VTE)

NOACs in the elderly*:* Pathak et al. demonstrated that the risk for VTE or VTE-related mortality in elderly patients with elective post-THR or TKR was similar with NOACs compared to LMWH (OR 0.62, 95% CI 0.30-1.26; p = 0.18; I = 44%), but the risk for bleeding was significantly lower (OR 0.71, 95% CI 0.53-0.94; p = 0.02; I = 0%) [2].

NOACs in obese patients*:* In a meta-analysis, efficacy of NOACs was similar to LMWH in preventing VTE and VTE-related mortality after arthroplasty in both overweight and obese patients (OR 0.64, p = 0.19 vs. OR 0.76, p = 0.43, respectively) without any difference in the risk of major bleeds compared to LMWH [2] and showed a trend of lower bleeding in obese patients (OR 0.83, p = 0.54, and OR 0.44, p = 0.07, respectively).

**Figure 4:**
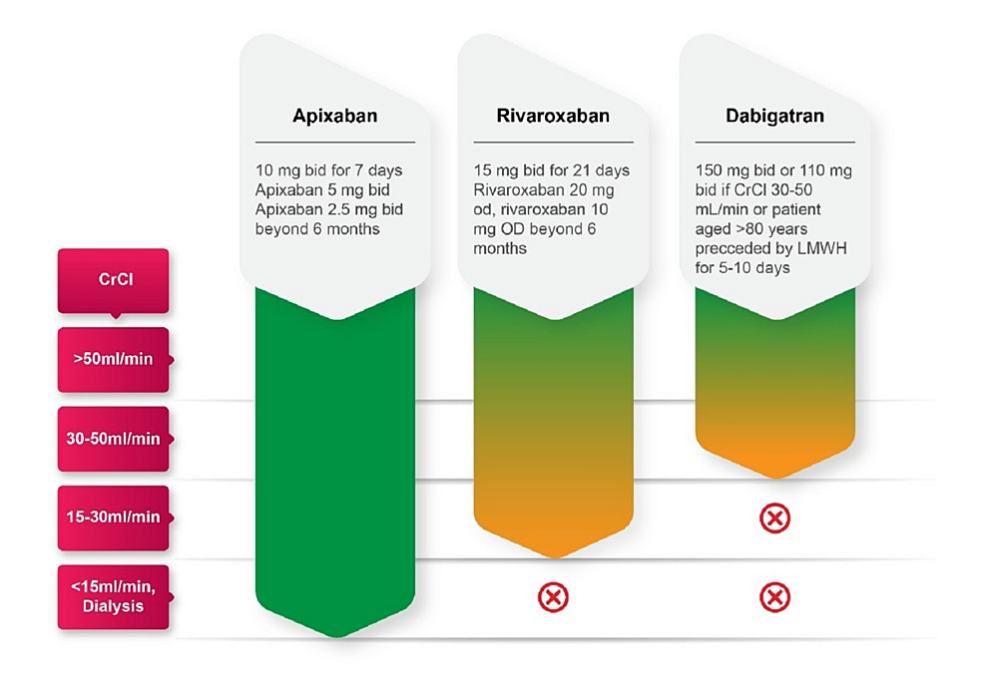
A summary of the dose of NOAC as per the renal function [69] NOAC: non-Vitamin K antagonist oral anticoagulant; bid: twice daily; CrCl: creatinine clearance; LMWH: low molecular weight heparin

VTE prophylaxis in patients with chronic kidney disease (CKD) undergoing TKR/THR:* *NOACs were associated with better efficacy in early CKD and similar efficacy and safety outcomes to warfarin in patients with CKD stages 4-5 or dialysis patients as reported by a meta-analysis [67]. All NOACs are partially eliminated by the kidneys. Dabigatran has the greatest dependence on renal elimination (88%) whereas renal elimination is 27% and 33% for apixaban and rivaroxaban, respectively. Therefore, renal impairment may alter renal elimination of the NOACs impacting the NOAC exposure. Renal impairment had no evident effect on the relationship between apixaban plasma concentration, so no dose adjustment is recommended for patients with renal impairment, including those with end-stage renal disease (ESRD) on dialysis and anti-FXa activity (Figure 4) [68]. In January 2021, there was a label update for apixaban in India for its use in patients even withCrCL<15ml/min and/or dialysis for all approved indications in the recommended doses.

NOACs may be the better choice in patients with a previous history of bleeding, a previous history of VTE, patients undergoing revision surgery, or surgery expected to last for more than two hours with extensive blood loss. Although there is no direct evidence for guiding NOAC use in these patients, there were fewer incidences of bleeding reported for these subgroups of patients as reported in all landmark randomized trials of NOACs.

Practical aspects of VTE prophylaxis post joint replacements in India

Assessment of Risk Factors: Thrombotic Versus Bleeding

Unfortunately, there are no VTE or bleeding risk assessment models (RAM) that have been sufficiently validated in patients undergoing orthopedic surgeries [3,69-71]. Hence, patients undergoing TKR or THR should receive VTE prophylaxis with an agent that offers an appropriate balance between thrombotic and bleeding risk. The factors that increase the risk of VTE and bleeding following total joint replacement are summarized in Table 4.

**Table 4:** Risk factors for VTE and bleeding development following THR and TKR Adapted from Kahn SR et al., Toth P et al., and Falck-Ytter Y et al. [3]. VTE: venous thromboembolism; TKR: total knee replacement; THR: total hip replacement

Risk factors for VTE
Previous VTE
Cardiovascular disease
Charlson comorbidity index ≥ 3
BMI > 25 kg/m^2^
Family history of VTE
Older age (per 5 years increase vs age <40 years)
Age ≥85 years
Varicose veins
Ambulation before postoperative day 2
Previous VTE or thrombophilia
Cancer chemotherapy
Immobilization
Oral contraceptives
Gender-female
Bilateral surgery
Surgery time >2years
Varicose veins
Risk factors for bleeding
Age >75 years
Previous major bleeding (and previous bleeding risk similar to current risk)
Chronic renal failure or hepatic disease
Low body weight
Concomitant antiplatelet agent
Surgical factors: history of or difficult-to-control surgical bleeding during the current operative procedure, extensive surgical dissection, and revision surgery

Initiating NOAC After Surgery: The Importance of Timing of Drug Initiation

Clinical trials evaluating NOACs have used varying timings of drug initiation after surgery. In the ADVANCE trials, apixaban was initiated 12-24 hours post-surgery, and in the RECORD trials, rivaroxaban was started six to eight hours postoperatively; whereas, in RE-MODEL and RE-NOVATE studies, dabigatran was initiated at half-dose only four hours post-surgery. Both the molecules have instructed in their labels to assess the hemostasis before initiating anticoagulants. Hemostasis would require at least about eight hours after surgery. In a comprehensive review of the timing of the first postoperative dose of anticoagulants, the authors suggested that based on the results of phase III trials with NOACs for thromboprophylaxis, their acceptable efficacy and safety can be achieved when an appropriate first postoperative dose of anticoagulant is delayed for at least six hours after surgery [72].

Perioperative Management of NOACs

**Figure 5:**
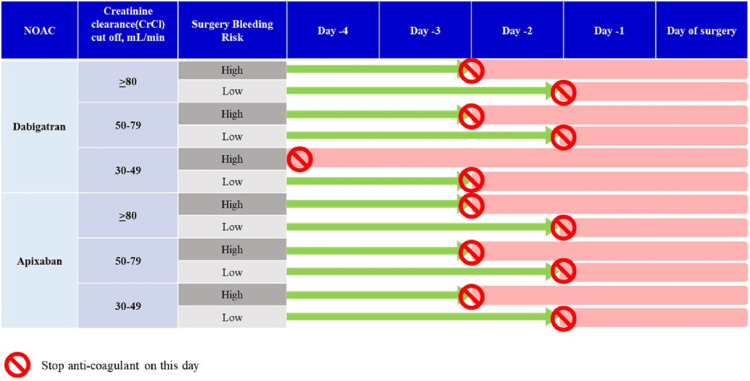
Peri-operative use of NOACs - elective procedure NOACs: non-Vitamin K antagonist oral anticoagulants [14]

Patients with atrial fibrillation (AF) or VTE who are already on NOACs must be advised to stop the anticoagulants before the surgery. Perioperative management of NOACs is relatively simpler compared to warfarin, owing to their shorter half-lives, shorter interruption intervals, and no need for laboratory monitoring. The exact timing for NOAC interruption depends on the drug’s half-life and the patient’s renal function. There is no universal consensus regarding the timing of discontinuation, but it is generally discontinued 24 hours (about two to three half-lives) before low-risk surgeries and two to four days before high-risk surgeries in patients with normal renal function (Figure 5) [14]. For elective surgeries, a blood coagulation panel is performed to estimate the risk of surgery, and a reversal is suggested only after a repeat coagulation panel. For immediate procedures, specific management depends on the level of urgency.

For re-initiating NOACs, the ACCP recommends re-initiating therapy 24-72 hours postoperatively (depending on the patient’s bleeding risk) to achieve wound hemostasis. NOAC interruption generally does not require bridging due to their short half-lives. In a scenario where a patient is at high postoperative bleeding risk, needs another procedure, or cannot yet tolerate oral medications, the American College of Cardiology (ACC) guidelines propose that unfractionated heparin (UFH) or LMWH may be temporarily used to bridge to a NOAC, though this is an uncommon scenario. Re-initiation of dabigatran is recommended one to four hours post-surgery, for rivaroxaban re-initiation is recommended six to ten hours post-surgery, and for apixaban, re-initiation is recommended 12-24 hours post-surgery. The 12-24 hours initiation window gives more time for establishing hemostasis following the procedure. Furthermore, initiation morning after the procedure diminishes the chances of the patient experiencing nausea and vomiting.

How to Manage Bleeding in Patients on NOAC: Should the Antidote Drive Selection of Anticoagulants?

Although NOACs are effective, the monitoring and reversal of their effect are critical challenges in clinical practice. There are two reversal agents currently available and are approved by the United States Food and Drug Administration (USFDA). Idarucizumab is the reversal agent for dabigatran and andexanet alfa is the reversal agent for Factor Xa inhibitors and they were approved by the FDA in 2015 and 2018, respectively. However, adexanet alfa is not yet available in India [73]. Current ACC guidelines recommend using these agents to manage bleeding associated with NOACs only in severe and life-threatening bleeding. The ACC recommends using prothrombin complex concentrate (PCC) during the unavailability of these agents. In fact, in a recent meta-analysis, [74] the effective hemostasis rate was shown similarly for all the reversal agents considered: prothrombin complex concentrates, idarucizumab or andexanet. If the risk of bleeding is anticipated, it is suggested that a VTE prophylactic agent with better safety and uncompromised efficacy should be used. NOACs have been reported to cause lesser major bleeding requiring antidotes compared to other standard-of-care anticoagulants, as reported in the RCTs. Therefore, antidote availability should not ideally drive the selection of NOAC.

Adherence to NOACs and Once-daily Versus b.i.d Dosing

In a meta-analysis, the b.i.d dosing regimen of non-VKA oral anticoagulants (direct thrombin inhibitors or factor Xa inhibitors) showed a more balanced risk-benefit profile for stroke prevention and intracranial hemorrhage in patients with atrial fibrillation [63]. Conversely, in another meta-analysis, once daily and b.i.d regimens did not show any difference in efficacy and safety of NOACs in major orthopedic surgery (MOS), non-valvular atrial fibrillation (NVAF), VTE, and acute coronary syndrome (ACS) [64]. Given that NOACs have plasma half-lives of approximately 12 hours, compared to once-daily dosing, b.i.d dosing may be associated with a lower probability of hazardously high peaks or low troughs when skipping a dose or when an overdose occurs [65].

Post-discharge Occurrence of VTE and Patient Follow-up

Important consideration must be given to the timing of most VTE events. The median length of hospital stay for THA or TKA outside the United States is 10 days, whereas it is three to seven days for the majority of Indian centers. Most VTE occurs after post-op day four. The Global Orthopedic Registry showed that the mean time for a symptomatic VTE event was 21.5 days for THA and 9.7 days for TKA. In an Asian Korean study, 20%-35% of VTE occurred after hospital discharge [75]. Thus, with the current trend towards shorter hospital stays, the majority of VTE events occur in outpatient settings, thus influencing the choice of anticoagulation prophylaxis [16,76]. Furthermore, the risk of wound complications like ecchymosis around the surgical wound, and hidden bleeding remains unaddressed in clinical trials. Residual blood in the joint, hemolysis, and tissue extravasation were the forms of hidden blood loss. Evidence has pointed to the idea that the administration of rivaroxaban has resulted in a higher hidden blood loss and a BTR (Blood transfusion rate). Patient follow-up after the surgery remains a great challenge in India. If patients experience any bleeding or VTE events after discharge post-TKR/THR, they usually consult either a physician or a cardiologist which makes it challenging for orthopedic doctors to keep a record of such patients. Therefore, it is imperative to choose a NOAC which has balanced safety or efficacy for VTE prophylaxis post-TKR/THR.

## Conclusions

The NOACs address many shortfalls of traditional agents and have become a crucial part of the armamentarium for thromboprophylaxis after THA and TKA. Available evidence shows that NOACs are as effective as LMWHs in preventing VTE. A dose-response-based meta-analysis has also shown lower bleeding for NOACs. Oral administration and the absence of dose monitoring make them easy to use in outpatient settings when compliance with treatment may be a challenge. They can be used after discharge for four to six weeks after TKA and THA, covering the at-risk period when most VTE events are reported. Current guidelines do not agree with recommending a preference for specific thromboprophylaxis agents. The use of aspirin remains controversial as most evidence is reported from low-quality observational research. Given the lack of evidence for a head-to-head comparison of NOACs, an agent with proven efficacy and safety may be preferred in special populations (elderly, obese patients, and those with renal dysfunction).
